# The growing importance of lone star ticks in a Lyme disease endemic county: Passive tick surveillance in Monmouth County, NJ, 2006 – 2016

**DOI:** 10.1371/journal.pone.0211778

**Published:** 2019-02-12

**Authors:** Robert A. Jordan, Andrea Egizi

**Affiliations:** 1 Tick-Borne Disease Program, Monmouth County Mosquito Control Division, Tinton Falls, New Jersey, United States of America; 2 Center for Vector Biology, Department of Entomology, Rutgers University, New Brunswick, New Jersey, United States of America; University of North Dakota School of Medicine and Health Sciences, UNITED STATES

## Abstract

As human cases of tick-borne disease continue to increase, there is a heightened imperative to collect data on human-tick encounters to inform disease prevention. Passive tick surveillance programs that encourage members of the public to submit ticks they have encountered can provide a relatively low-cost means of collecting such data. We report the results of 11 years of tick submissions (2006–2016) collected in Monmouth County, New Jersey, an Atlantic coastal county long endemic for Lyme disease. A total of 8,608 ticks acquired in 22 U.S. states were submitted, 89.7% of which were acquired in Monmouth County, from 52 of the County’s 53 municipalities. Seasonal submission rates reflected known phenology of common human-biting ticks, but annual submissions of both *Amblyomma americanum* and *Dermacentor variabilis* increased significantly over time while numbers of *Ixodes scapularis* remained static. By 2016, *A*. *americanum* had expanded northward in the county and now accounted for nearly half (48.1%) of submissions, far outpacing encounters with *I*. *scapularis* (28.2% of submissions). Across all tick species and stages the greatest number of ticks were removed from children (ages 0–9, 40.8%) and older adults (ages 50+, 23.8%) and these age groups were also more likely to submit partially or fully engorged ticks, suggesting increased risk of tick-borne disease transmission to these vulnerable age groups. Significantly more people (43.2%) reported acquiring ticks at their place of residence than in a park or natural area (17.9%). This pattern was more pronounced for residents over 60 years of age (72.7% acquired at home). Education that stresses frequent tick checks should target older age groups engaged in activity around the home. Our results strongly suggest that encounter rates with ticks other than *I*. *scapularis* are substantial and increasing and that their role in causing human illness should be carefully investigated.

## Introduction

Lyme disease (LD), caused by the bacterium *Borrelia burgdorferi*, is the most commonly reported vector-borne disease in the United States with most cases reported from areas of traditionally high incidence in the Northeast, mid-Atlantic, and upper Midwest regions where case rates have remained stable or decreased during the reporting period 2008–2015 [1]. However, the landscape of tick-borne diseases continues to change and expand [2], and cases of all tick-borne diseases are increasing [3]. In particular, an increase in spotted fever rickettsiosis cases and a decrease in severity of those cases has led to a reevaluation of which *Rickettsia* species are causing human disease and which tick species are transmitting them [4, 5]. Additional focus in recent years has centered on underrecognition of ehrlichiosis cases [6, 7] and emergence of new viruses [8] transmitted by the lone star tick, *Amblyomma americanum*. Many of these expanding and emerging disease agents have been identified in ticks collected from New Jersey [9, 10].

Data on vector distributions and rates of infection with pathogens (primarily *B*. *burgdorferi*) are easily derived from field collections of ticks and have been analyzed on an array of spatial scales and rendered as risk maps [11]. Conversely, the collection of adequate data for human infection with tick-borne pathogens or human encounters with infected ticks is more challenging. As a result, the density of infected ticks in the environment has been widely adopted as a surrogate for human risk of exposure [12]. Several studies have linked counts of host-seeking blacklegged ticks, *Ixodes scapularis*, and their rate of infection with *B*. *burgdorferi* to LD incidence [13–16]. More recently, Pepin et al. [11] demonstrated a positive correlation between density of infected nymphs in *I*. *scapularis* and LD across more than 300 locations in the Northeast and Midwest, but were unable to consistently predict LD cases using tick collection data in areas where LD was emergent.

However, the putative link between the abundance of infected questing ticks and risk of human illness has been questioned. Actual risk of infection is dependent on an array of factors beyond tick encounter rates such as human behavior and utilization of landscape features and the likelihood that a tick bite results in actual infection which is related to the duration of attachment [12].

A system of passive surveillance that offers free tick identification to the public can be an economical means of collecting data that directly links the distribution of ticks and pathogen infection prevalence to concurrent human–tick encounters and the incidence of human tick-borne diseases at a variety of spatial scales. Previous studies have demonstrated a positive relationship between passive surveillance submissions of *I*. *scapularis* removed from people and LD cases in both LD endemic areas and in areas of emerging disease [17, 18]. Passive surveillance has been used to characterize the expanding range and apparent proliferation of *I*. *scapularis* populations in Canada [19, 20], describe *Dermacentor variabilis* range expansion in Maine [17] and suggest the potential for *A*. *americanum* expansion into Massachusetts [21].

Passive tick surveillance has been conducted on fairly large spatial scales in New York [22], Maine [17], Canada [19, 20], Iowa [23], Massachusetts [21], and across the eastern U.S. by the U.S. military [24]. The relationship between the abundance of *B*. *burgdorferi*-infected *I*. *scapularis* nymphs and LD incidence, however, is strongly affected by the spatial scale of measurement and appears to be strongest at the scale of community or county [12]. We are unaware of any previous detailed analysis of passive surveillance data collected at the scale of an individual county in a long-term endemic area.

Here we present analysis of 11 years of passive tick surveillance data from a LD endemic county in New Jersey, including (1) relative numbers of submissions of 3 medically-important tick species: *I*. *scapularis*, *A*. *americanum*, and *D*. *variabilis*; (2) their spatial distribution, seasonal phenology, and long-term trends in encounter rates as revealed by tick submissions; (3) demographic characteristics of human hosts of the three tick species; and (4) *B*. *burgdorferi* infection prevalence in submitted *I*. *scapularis* and relationships with reported county LD case rates. We discuss the utility of passive surveillance data for monitoring human risk in a changing tick-borne disease landscape and suggest strategies for additional research and education efforts informed by these data.

## Materials and methods

### Site description and ethics statement

Monmouth County (40°44′N, 74°17′W) is located in eastern-central New Jersey, a US state on the mid-Atlantic coast. The county is 468.8 mi^2^ in size and had a population of 630,380 (1,344.7 persons/mi^2^) as of the 2010 census (http://www.census.gov/quickfacts/table/PST045215/34025).

Monmouth County is bisected by the geomorphologic break that separates the Inner and Outer Coastal Plain physiographic provinces, resulting in an array of habitat types that support tick populations, often in close proximity to residential areas [25–28]. The county comprises 53 municipalities and several large natural areas/parks. Historically, the first cases of LD in Monmouth County were diagnosed in 1979 [29] and numbers have continued to increase over time, reaching several hundred cases of LD annually with an 11-year average (2006–2016) of 396 (± 89.2SD) cases/year (a case rate of 62.57 (± 14.2SD)/100,000) (https://www-doh.state.nj.us/doh-shad). In 2016, it was ranked 6^th^ highest nationally for LD cases among all US counties (https://www.cdc.gov/lyme/stats/index.html).

This study reports data from a surveillance program that is part of the regular duties of the Mosquito Control Division as designated by the Monmouth County Board of Chosen Freeholders (Resolution No. 97–468) therefore ethics committee approval was not required. Submissions were assigned a unique identification number to ensure that the data contained no personal identifiers.

### Tick collection and passive surveillance program

The Monmouth County Mosquito Control Division’s tick identification and testing service was initiated in 2005. The service allows county residents to submit ticks they have encountered and receive a report identifying the species, life stage, and status (flat, partially, or fully engorged) of the submitted tick. Residents were initially made aware of the program with a county-wide press release, and in subsequent years awareness was sustained by posting information about the program on the county website and providing literature to county fair attendees, school nurses, and physician offices. Species-level identification of ticks was based on published identification keys [30–32]. In addition, prior to 2017, the program also offered testing of adult female and nymphal *I*. *scapularis* for *B*. *burgdorferi* infection for a small fee (tick identification has always been free of charge).

Persons submitting ticks were asked to complete a form with the following information: date of the encounter, location of the encounter (municipality as well as home, park, school, etc.) and what the person was doing at the time (e.g., yard work, recreation, employment, etc.). In addition, they were asked to provide the age, gender and species of the host, where on the host’s body the tick was attached, and an estimate of the length of time the tick had been attached. There was no enforced requirement to complete the entire form and occasionally residents were unable to remember or unsure of where they encountered the tick; therefore, not all forms were completed fully or with absolute accuracy.

The following analysis and discussion considers ticks submitted to the program from January 2006 (the first complete year of the program) through December 2016 (the last year that *B*. *burgdorferi* testing was offered).

### Detection of pathogen DNA in *I*. *scapularis* by PCR

From 2006–2014, DNA was isolated from *I*. *scapularis* ticks using DNAzol^®^ Genomic DNA isolation reagent (Molecular Research Center, Inc., Cincinnati, OH), amplified with *B*. *burgdorferi* PCR primers FLA1/FLA2 and visualized on an electrophoresis gel, as previously described [33]. In 2015–2016, DNA was extracted from *I*. *scapularis* ticks using Qiagen DNeasy Blood & Tissue kits (Qiagen Inc, Valencia, CA) and they were tested for *B*. *burgdorferi* using a TaqMan real-time PCR assay [34]. Additionally, during the years 2006–2014 entire engorged and partially engorged ticks were extracted, whereas during 2015–2016, due to concerns about possible PCR inhibition by blood components, only the head and anterior 1/4 abdomens of engorged ticks were extracted. The positive control for *B*. *burgdorferi* (ATCC 35210D) was purchased from American Type Culture Collection, Manassas, VA.

### Data analysis

Statistical analyses were limited to ticks acquired within Monmouth County, as reported on submission forms (7,722 specimens or 87% of submissions). Tick species that were only submitted once in the 11-year period were also excluded. Where indicated, data were transformed to meet assumptions of normality.

Annual submissions of each species were log-transformed and examined for trends over time using simple linear regression. The length of the active questing season, defined as the period between the dates of first and last submission for each year, was contrasted between 2006–2011 and 2012–2016 submission periods across tick species and stages using Mann-Whitney U-tests or Kruskal-Wallis multiple comparisons tests. Chi-square contingency tests were used to compare location of attachment of submitted ticks between species and stages, numbers of infected ticks between fed and flat ticks, and numbers of ticks acquired between different locations and during different activities. Annual trends between numbers of tick submissions and Lyme disease cases were analyzed using simple linear regression on natural log-transformed data, while annual trends in B. burgdorferi infection prevalence (% infected) were analyzed using arcsine-transformed values. After analysis showed no autocorrelation for series (Durbin-Watson test, P > 0.05), relationships between transformed numbers of submissions, numbers of infected ticks and infection prevalence rates were explored using Pearson correlation coefficients. All statistical tests were performed using STATISTICA 5 (StatSoft, Tulsa, OK).

Maps of tick submissions were created in QGIS using an open source layer for US municipalities (https://qgis.org/). The complete dataset is available in S1 Table.

## Results

### Geographical and temporal distributions of tick species

**Fig 1:**
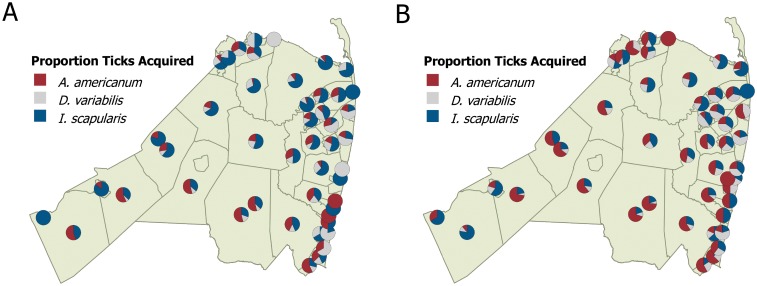
Species composition of ticks submitted in Monmouth County, by acquired municipality. A) 2006–2011, B) 2012–2016.

During the 2006–2016 study period, we received a total of 8,608 ticks acquired in 22 U.S. states. Of those, a total of 8,280 (96.2%) ticks were acquired in New Jersey from all of New Jersey’s 21 counties. The following analyses are restricted to ticks acquired within Monmouth County (89.7%). A total of 7,722 submitted ticks included 3,105 (40.2%) *I*. *scapularis*, 3,042 (39.4%) *A*. *americanum*, and 1,575 (20.4%) *D*. *variabilis*, and one each *I*. *dentatus*, *I*. *cookei*, and *Rhipicephalus sanguineus*. A total of 5,693 submission forms included the municipality where the tick was acquired (74.7%) and ticks were acquired in 52 of Monmouth County’s 53 municipalities (Fig 1).

The distribution of submissions by developmental stage (adult, nymph, larva) varied among the three species (Table 1). While *I*. *scapularis* submissions were split roughly 50–50 between adult females and nymphs, fully 61% of all *A*. *americanum* submissions were nymphal ticks. Only two (0.1%) of the *D*. *variabilis* submissions were nymphs, as immatures of this species feed mainly on non-domestic rodents and Carnivora, and are unlikely to be encountered by humans.

**Table 1:** Distribution of tick submissions by developmental stage and species.

Stage	Tick Species					
	*I*. *scapularis*		*A*. *americanum*		*D*. *variabilis*	
	N	%	N	%	N	%
Female	1575	50.7%	416	13.7%	886	56.3%
Male	24	0.8%	576	18.9%	687	43.6%
Nymph	1415	45.6%	1856	61.0%	2	0.1%
Larva	91	2.9%	194	6.4%	0	0.0%
Total	3105		3042		1575	

**Fig 2:**
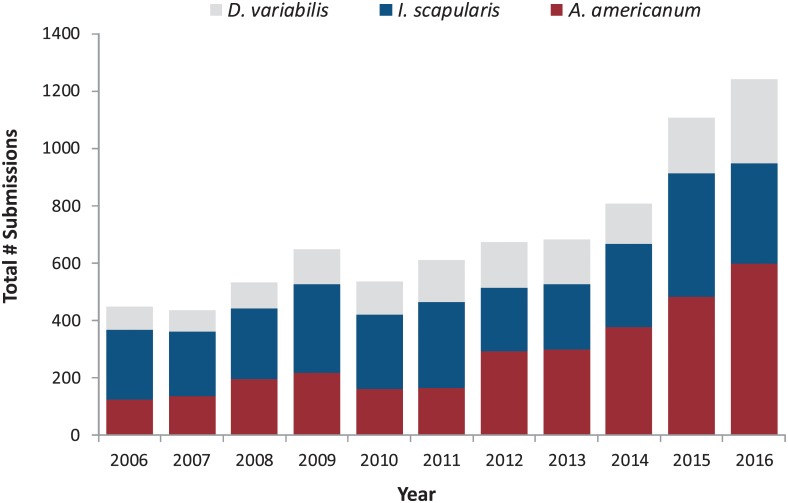
Total annual submissions comprising 3 medically-important tick species, 2006–2016.

The species composition of annual total submissions has changed substantially over time (Fig 2). Total submissions (all stages) of both *A*. *americanum* and *D*. *variabilis* increased over the period (*R*^2^ = 0.82, *P* < 0.01 and *R*^2^ = 0.76, *P* < 0.01, respectively), while the trend in *I*. *scapularis* submissions was weaker (*R*^2^ = 0.37, *P* = 0.047) and reflects a substantial one year increase in *I*. *scapularis* submissions in 2015 (without 2015: *R*^2^ = 0.26, *P* = 0.13). For nymphal ticks specifically, submissions of *A*. *americanum* comprised an increasing proportion of total submissions over time (*R*^2^ = 0.78, *P* < 0.01), while numbers of nymphal *I*. *scapularis* remained relatively static (*R*^2^ = 0.29, *P* = 0.05).

As a result, while *I*. *scapularis* made up the majority of submissions in the years prior to 2012 (mean ± SD = 49.5% ±3.0 for *I*. *scapularis* vs. 30.9% ±3.9 for *A*. *americanum*), during the 2012–2016 period, *A*. *americanum* became the most commonly submitted tick (45.1% ±2.1 for *A*. *americanum* vs. 33.9% ± 4.0 for *I*. *scapularis)*. This shift coincided with a marked increase in *A*. *americanum* submissions from several municipalities in the northern and western parts of the county (Fig 1).

**Fig 3:**
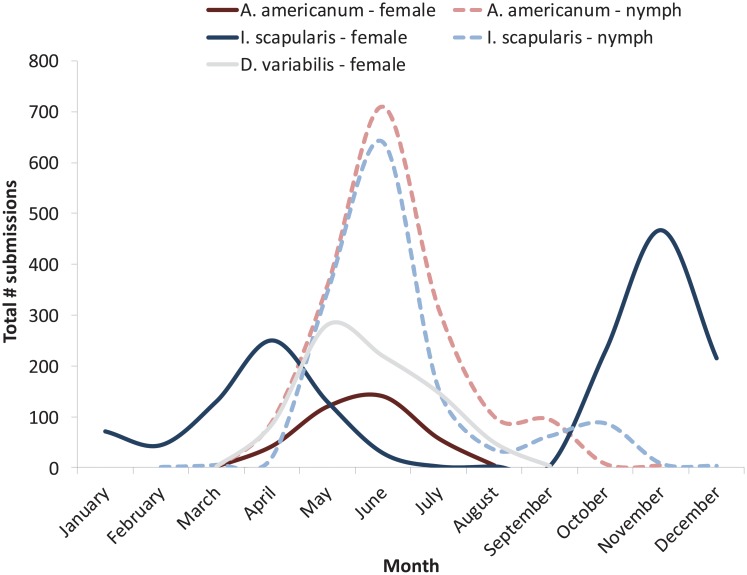
Seasonal phenology of 3 medically-important tick species reflected in passive surveillance submissions. Monmouth County, New Jersey, 2006–2016. Only 2 *D*. *variabilis* nymphs were submitted, both during the month of June.

*I*. *scapularis* submissions were received in all months of the year while *A*. *americanum* and *D*. *variabilis* submissions were generally restricted to spring and summer months. Developmental stages exhibited different seasonal peaks that reflected established phenology of tick species in New Jersey and Monmouth County (Fig 3). We observed two distinct peaks in submissions of adult *I*. *scapularis*: an early spring peak representing emergence of overwintering cohorts of ticks from the previous year and a larger peak in October-December representing the larger fall populations of questing adults previously characterized in New Jersey [25]. We also observed a well-described peak in nymphal *I*. *scapularis* submissions during May-June, and a second smaller peak in nymphal submissions (consistent across years) in August-September (Fig 3).

The mean length of the *A*. *americanum* nymphal season (defined as date of first submitted nymph to date of last submitted nymph) increased dramatically from 163.5 ± 25.8 (Mean ± SD) days during 2006–2011 to 294.8 ± 136.6 days during 2012–2016 (Mann-Whitney *U*_(6,5)_ = 3.00, P = 0.03, medians = 157 and 218, respectively). This increase was not observed for *I*. *scapularis* nymphs (mean of 192.7 ± 28.0 days vs. 196.6 ± 23.0 days, medians 192 and 203, respectively) (*U*_(6,5)_ = 13.00, P = 0.72)),or for adults of *I*. *scapularis* (237.0 ± 12.7 vs. 242.2 ± 12.2 days, U_(6,5)_ = 12.00, P = 0.58, *A*. *americanum* (133.7 ± 15.2 vs. 141.2 ± 13.3 days, U_(6,5)_ = 10.00, P = 0.36) or *D*. *variabilis* (141.2 ± 25.0 vs. 159.8 ± 35.2 days, U_(6,5)_ = 11.00, P = 0.54).

### Human host demography and tick feeding patterns

A total of 7,297 (94.5%) ticks were removed from human hosts, 364 (4.7%) from dogs, 20 (0.3%) from cats, 11 (0.1%) from other pets, 16 (0.2%) were found unattached, and the remainder did not report a host. Of the ticks removed from human hosts where sex of host was reported, 7,200 (98.7% of all submissions), 52.7% came from men and 47.3% came from women.

Age of host was reported for 5,868 (80.4%) of submitted ticks. Human host age distribution varied among tick species and developmental stages (Table 2). Host age data was obtained for 2,863 *I*. *scapularis* (1,473 female ticks, 1,390 nymphs), 2,183 *A*. *americanum* (395 female ticks, 1,788 nymphs), and 822 *D*. *variabilis* (319 female ticks). Across all species and stages the greatest number of ticks were removed from children (ages 0–9, 40.8%) although *A*. *americanum* was removed from older hosts more often than *D*. *variabilis* or *I*. *scapularis* (53.2% of ticks from people > 50 yrs were *A*. *americanum*) (Table 2).

**Table 2:** Percentage and rate of tick submissions by human host age classes. Monmouth County population data obtained from U.S. Census Bureau [35].

		*I*. *scapularis*				*A*. *americanum*				*D*. *variabilis*	
Host Age	Pop (%)	Nymphs		Adult Females		Nymphs		Adult Females		Adult Females	
		N (%)	Rate	N (%)	Rate	N (%)	Rate	N (%)	Rate	N (%)	Rate
<10	11.0	742 (54.0)	1077.8	499 (38.2)	724.8	601 (33.8)	873.0	103 (27.7)	149.6	402 (51.5)	583.9
10–19	13.0	200 (14.5)	245.8	189 (14.5)	232.3	195 (11.0)	239.7	41 (11.0)	50.4	110 (14.1)	135.2
20–29	12.0	29 (2.1)	38.6	45 (3.4)	59.9	54 (3.0)	71.9	9 (2.4)	12.0	14 (1.8)	18.6
30–39	11.0	97 (7.1)	140.9	84 (6.4)	122.0	136 (7.6)	197.6	27 (7.3)	39.2	28 (3.6)	40.7
40–49	13.0	109 (7.9)	134.0	153 (11.7)	188.1	251 (14.1)	308.5	51 (13.7)	62.7	77 (9.9)	94.6
50–59	17.0	80 (5.8)	75.2	161 (12.3)	151.3	242 (13.6)	227.5	65 (17.5)	61.1	67 (8.6)	63.0
60–69	12.0	75 (5.5)	99.9	120 (9.2)	159.8	177 (10.0)	235.7	48 (12.9)	63.9	44 (5.6)	58.6
70–79	7.0	33 (2.4)	75.3	45 (3.4)	102.7	108 (6.1)	246.5	24 (6.5)	54.8	23 (2.9)	52.5
≥80	4.0	10 (0.7)	40.0	11 (0.8)	43.9	14 (0.8)	55.9	4 (1.1)	16.0	16 (2.0)	63.9

Pop = percentage of Monmouth County population in each age class.

Rate = Number of ticks submitted/100,000 persons in Monmouth County.

Location of attachment varied significantly by species (χ^2^ = 1609.83, df = 8, *P* < 0.01) (Table 3). Greater numbers of *D*. *variabilis* adults (as compared to nymphs and adults of the other 2 species) were reported to have been removed from the head of people, while significantly more *A*. *americanum* nymphs and adult females were removed from the lower body. The distribution of *I*. *scapularis* ticks generally tended to be more evenly distributed across the body (Table 3).

**Table 3:** Reported location of attachment on human hosts.

Tick Species	Location				
	Head N (%)	Arms N (%)	Upper Torso N (%)	Lower Torso N (%)	Legs N (%)
*I*. *scapularis*	596 (22.0)	325 (12.0)	805 (29.7)	761 (28.1)	220 (8.1)
*A*. *americanum*	311 (11.8)	149 (5.7)	727 (27.6)	1145 (43.5)	302 (11.4)
*D*. *variabilis*	882 (66.6)	56 (4.2)	208 (15.7)	101 (7.6)	77 (5.8)

Upper torso = chest and back, Lower torso = buttocks and groin.

The likelihood that a submitted tick was flat (no evidence of feeding) or fed (partially or fully engorged) varied among species: significantly fewer *A*. *americanum* females removed from human hosts had taken at least a partial blood meal as compared to adult females of the other 2 species (*χ*^2^ = 21.23, df = 2, *P* < 0.01), while significantly more nymphal *A*. *americanum* were at least partially engorged, relative to *I*. *scapularis* nymphs (*χ*^2^ = 111.03, df = 1, *P* < 0.01) (Table 4).

**Table 4:** Degree of engorgement (fed vs. flat) of submitted ticks removed from human hosts.

		Degree of Engorgement		
	Species	Fed	Flat	% Fed
A. Females	*I*. *scapularis*	569	904	38.8
	*A*. *americanum*	105	290	26.6
	*D*. *variabilis*	319	503	38.6
B. Nymphs	*I*. *scapularis*	617	773	44.4
	*A*. *americanum*	1130	658	63.2

“Fed”–visually either partially or fully engorged; “Flat”–no visual evidence of having fed.

**Fig 4:**
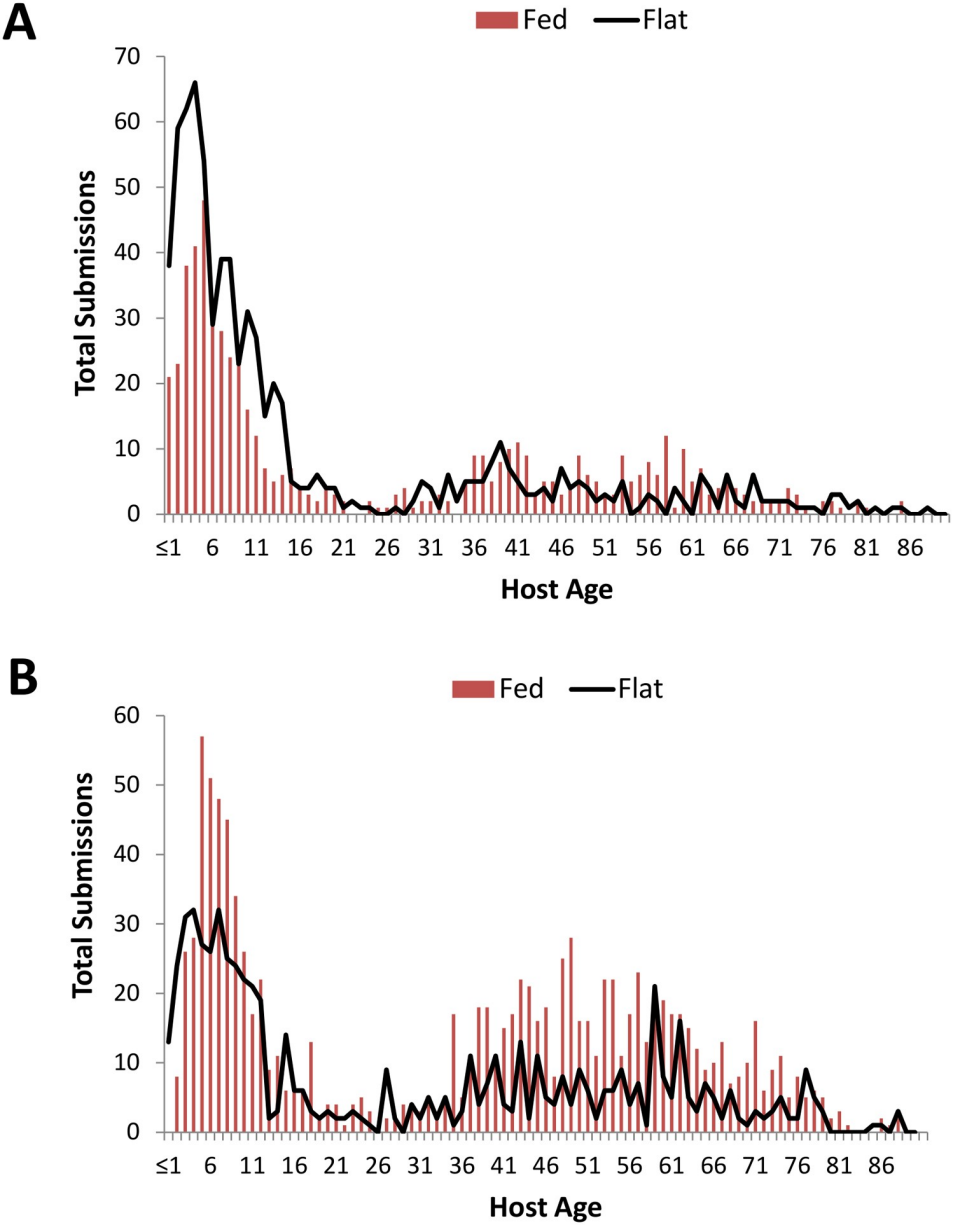
Differences in host age distribution of flat vs. fed ticks between tick species. A) *Ixodes scapularis*, B) *Amblyomma americanum*.

In particular, the distribution of fed and flat ticks among different human host age groups varied between *I*. *scapularis* and *A*. *americanum* nymphs (Fig 4). In general, more *A*. *americanum* nymphs were removed from older hosts and more *A*. *americanum* nymphs were either partially or fully engorged when removed from the youngest and oldest age classes. Percentage of fed nymphs was higher for *A*. *americanum* than *I*. *scapularis* in most host age classes.

### Location of tick encounters

A total of 5,646 (73.1%) of people submitting ticks provided information about where they think they acquired the tick they were submitting (Table 5). Responses were grouped into “home,” “park,” “school,” and “other”. Activities were grouped into “recreation,” “yard work,” “employment,” or “other.” Significantly more people (43.2%) reported acquiring the tick at their place of residence than in a park or natural area (17.9%). Significantly more people 10–29 years old reported acquiring ticks in parks or natural areas, while more people in older age classes (> 50 yr old) tended to encounter ticks peridomestically (χ^2^ = 190.69, df = 4, *P* < 0.01) (Table 5). While younger age groups (<29 yr) tended to report acquiring ticks during recreational activity, older individuals claimed to have acquired ticks while engaged in yard work or other property maintenance activity (χ^2^ = 1780.17, df = 4, *P* < 0.01). In particular, older people (>60 yr) were most at risk at home (72.7% of submission reporting an activity) and while engaged in yard work (61.8% of submissions).

**Table 5:** Reported location and activity where ticks were acquired.

Host Age	Location Acquired		Activity Acquired	
	Home N (%)	Park N (%)	Recreation N (%)	Yardwork N (%)
<10	1240 (67.9)	586 (32.1)	2229 (97.4)	60 (2.6)
10–29	314 (46.3)	364 (53.7)	813 (93.9)	53 (6.1)
30–49	627 (67.6)	301 (32.4)	570 (61.9)	351 (38.1)
50–69	681 (72.8)	254 (27.2)	386 (44.7)	477 (55.3)
≥70	229 (86.4)	36 (13.6)	55 (22.3)	192 (77.7)

### *Borrelia burgdorferi* infection prevalence in *I*. *scapularis* removed from human hosts

Ninety-six percent of submitters of *I*. *scapularis* ticks chose to have their ticks tested. We tested 1,517 adult female and 1,391 nymphal *I*. *scapularis* ticks acquired in Monmouth County 2006–2016 for *B*. *burgdorferi* infection (Table 6). Flat adult females had higher rates of infection than fed females (χ^2^ = 45.51, df = 1, P < 0.01), but engorgement status had no effect on nymphal infection rates (χ^2^ = 3.23, df = 1, P = 0.07). A similar pattern has been observed in prior studies [36] and could relate to the presence of PCR inhibitors in blood, of which females take a much larger quantity than nymphs. Mean annual infection prevalence 2006–2016 in adult females (38.2 ± 0.06) and nymphs (22.4 ± 0.04) did not vary over time during the 11 year period (*R*^*2*^ = 0.1103, P = 0.32 for adults and *R*^*2*^ = 0.0477, P = 0.52 for nymphs).

**Table 6:** *Borrelia burgdorferi* infection rates in flat vs. fed *I*. *scapularis* submitted 2006–2016.

		*B*. *burgdorferi* infection prevalence		
	Engorgement level	Positive	Negative	% Pos
A. Females	Flat	405	492	45.2
	Fed	173	447	27.9
	Total	578	939	38.1
B. Nymphs	Flat	158	613	20.5
	Fed	153	467	24.6
	Total	311	1080	22.4

Infection prevalence did not differ significantly between adult female *I*. *scapularis* submitted in the fall (mean = 39.5 (±7.3SD), range = 25.7–50.0, median = 39.5) and spring (March-April) (mean = 38.6 (±8.4SD), range = 21.6–50.7, median = 37.8) as over-wintering adults (Mann-Whitney *U*_(11,11)_ = 57.0; *P* = 0.82).

### Relationship between *I*. *scapularis* infection prevalence and Monmouth County Lyme disease incidence

**Fig 5:**
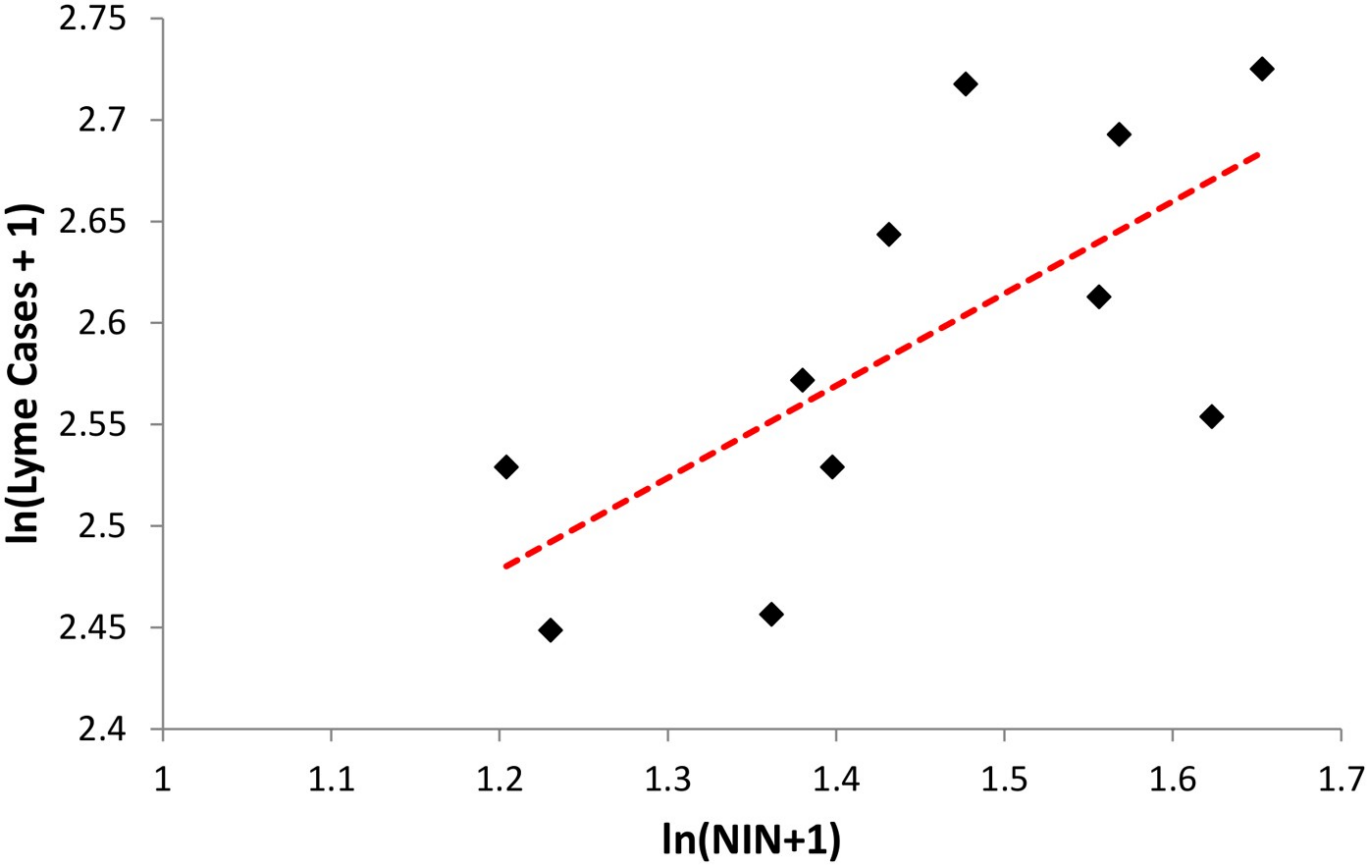
Relationship between number of infected *I*. *scapularis* nymphs (NIN) and Monmouth County Lyme disease cases, 2006–2016.

Between 2006 and 2016, 4,359 cases of LD were reported by Monmouth County residents (mean annual case rate of 62.57/100,000). After analysis showed no autocorrelation for series (P > 0.05), we examined correlation between numbers of LD-infected *I*. *scapularis* nymphs and reported LD case rates for Monmouth County and total tick submissions over time using Pearson correlation coefficients [37]. There was a significant positive correlation between annual reported LD cases in Monmouth County and numbers of infected nymphal *I*. *scapularis* submitted during 2006–2016 (*r* = 0.67, *P* = 0.026) (Fig 5). LD cases also correlated with total numbers of nymphal *I*. *scapularis* (*r* = 0.60, *P* = 0.050), nymphal infection prevalence in *I*. *scapularis* (*r* = 0.61, *P* = 0.046) and interestingly, with total ticks submitted of all species (*r* = 0.71, P = 0.015).

## Discussion

Passive tick surveillance data collected in a single LD endemic county presented here provides a picture of the changing tick-borne disease landscape over an 11 yr period, with implications for state and region-wide disease surveillance. We observed a dramatic change in the species composition of tick submissions over time, with submissions of both *A*. *americanum* and *D*. *variabilis* increasing over the period 2006–2016 while *I*. *scapularis* submissions remained comparatively flat. Subadult *A*. *americanum* have become the most common tick submitted, suggesting increased human exposure to *A*. *americanum* ticks relative to *I*. *scapularis* ticks active at the same time of year. The length of time from first to last submission of nymphal *A*. *americanum* has also lengthened in recent years, indicating a larger portion of the year during which human exposure can occur.

The geographic distribution of *A*. *americanum* in Monmouth County was previously believed to be restricted to the southern portion of the county where hydrologic and edaphic conditions and the resulting vegetation types are typical of the Atlantic coastal pine forest habitat characteristic for this species [10]. Nevertheless, results presented here show that *A*. *americanum* is now sympatric with *I*. *scapularis* throughout much of the county and their immature stages share a similar seasonal phenology. Prior field studies have suggested that *A*. *americanum* is able to successfully establish populations in a broader range of habitats than *I*. *scapularis* [26, 38]. Where they co-occur, *A*. *americanum* is far more aggressive, attacking human hosts in all life stages (adult, nymph, and larvae) and are typically by far the more numerous of the ticks in the same habitats [27]. Consequently, given the abundance and aggressive host-seeking behavior of *A*. *americanum* ticks, it is not surprising to see high rates of human encounters with them [27, 28, 39].

Geographic ranges of both *A*. *americanum* and *D*. *variabilis* have expanded in different parts of the United States in recent years [40] and both are implicated in the changing epidemiology of spotted fever group rickettsioses [5]. While human serology is unable to distinguish the exact pathogen species (and therefore the vector) responsible, growing numbers of spotted fever rickettsiosis cases within Monmouth [41] echo our finding that human encounters with both species are increasing. Human cases of *A*. *americanum*-transmitted ehrlichiosis are also on the rise in the US [42] and in Monmouth County, although potentially many more go undetected [6].

Tick-borne disease risk encompasses not only the activity of tick populations, but also the effects (or lack thereof) of preventive human behaviors. Overall we observed that young children (0–9) and older adults (50+) made up a greater proportion of hosts for submitted ticks compared with young and middle-aged adults. Other studies have observed similar patterns. Rand et al. [17] reported high numbers of *I*. *scapularis* ticks removed from children below 15 yr of age, while few ticks were removed from people in their late teens and twenties. This could be due to a combination of factors including differences in exposure to tick habitat, host behaviors in different habitats (e.g., yard work), willingness to submit ticks, and awareness, among others [43]. For example, small children may play in areas closer to where ticks normally quest while young adults may spend more time on indoor activities that limit exposure to ticks [17]. Alternatively, concerned parents may be more likely to submit ticks from young children whereas young adults tend to be more accepting of tick-borne disease risk [44].

In addition to age-specific differences in overall tick exposure, we also observed differences in the length of time ticks were attached before being removed in different age groups. Early tick detection and removal dramatically decreases the chance of contracting a tick-borne disease when a tick bite occurs [45] therefore our finding that ticks fed longer on young children and the elderly is concerning. Rand et al. [17] found that 18.9% of passively submitted *I*. *scapularis* ticks removed from people in their 70s were at least partially engorged, compared to ≈ 8% from people < 20 yr of age and only 1% from people in their 20s. They found this pattern held for both nymphal and (larger, presumably easier to see) adult ticks. Other studies of *I*. *scapularis* submissions have also demonstrated that older age groups are more likely to submit engorged or partially engorged ticks [20, 21]. These data indicate a need to target public health messaging on tick prevention and early removal to these vulnerable age groups, particularly as the proportion of the population 65 and older is projected to increase within Monmouth County, from 16% in 2010 to 22% in 2034, with similar trends reported in the US as a whole [46].

We report species-specific differences in the location of tick attachment on human hosts, with *D*. *variabilis* most often reported attached to the head and *A*. *americanum* most often reported from the lower torso, while *I*. *scapularis* did not appear to demonstrate a preference. Yet the significance of these differences is unclear and prior studies that analyzed tick attachment site have presented inconsistent results. Felz and Durden [47] found no apparent preference for attachment sites of four species of ticks on human hosts in Georgia and South Carolina, while Falco and Fish [22] found that nymphal *I*. *scapularis* in New York were found most often on lower extremities and adult ticks on the head. Xu et al. [21] found significant differences between nymphal and adult *I*. *scapularis* attachment sites: Nymphs were more likely to be attached to lower extremities, while adult ticks were more frequently found attached near the head, followed by the lower extremities.

In addition to understanding the demographic groups most at risk, prevention of tick-borne disease requires an understanding about where and when people are most likely to be exposed to infected ticks [43]. Significantly more people reported acquiring ticks at their place of residence than in a park or natural area. Previous studies have linked LD risk to peridomestic exposure to infected *I*. *scapularis* [16, 48–51] although other studies have suggested substantial exposure in other locations [22, 52]. Connally et al. [16] showed that simple density of infected ticks was inadequate as a predictor of LD risk in peridomestic settings without additional information about how people used those areas [43]. Further study is needed to understand how human activity and behavior influence tick encounters.

The link between seasonal abundance of questing *I*. *scapularis* nymphs and LD date of onset is well-established [1] and often, though not always, supported by correlations between human disease cases and numbers or infection levels of *I*. *scapularis* submitted to passive surveillance programs ([17, 21, 53], but see [54]). While we report a high positive correlation between the number of *B*. *burgdorferi*- infected *I*. *scapularis* nymphs (NIN) submitted and number of reported LD cases within Monmouth County, we observed an even stronger correlation between tick submissions of all species and reported LD cases. Rossi et al. [54] also reported an unexpected positive association between submission rates for *A*. *americanum* and *Dermacentor* spp. ticks and LD incidence, and for all tick species combined and LD incidence. This outcome may be related to the difficulty experienced by the public and clinicians in differentiating *I*. *scapularis* from other species and discriminating among tick-borne diseases presenting with similar symptoms. For example, Armstrong et al. [55] demonstrated that residents in areas where *A*. *americanum* was abundant and aggressively biting them tended to have an exaggerated perception of the risk of acquiring LD. Bites from *A*. *americanum* can cause an erythema migrans rash known as southern tick-associated rash illness (STARI) that could be incorrectly reported as LD in endemic areas [55, 56]. Recently, cases of STARI have been observed in New York and New Jersey [57, 58].

We observed little or no measurable variation in *B*. *burgdorferi* infection rates in passively collected *I*. *scapularis* nymphs and adult females. Xu et al. [21] also found that the prevalence of *B*. *burgdorferi* infection in passively collected *I*. *scapularis* nymphs and adults was relatively uniform among four areas of high tick density in Massachusetts, and that despite an increase in reported LD cases and tick numbers in recent years, there was little annual variation in the prevalence of *B*. *burgdorferi* infection among passively and actively collected ticks. By contrast, annually increasing prevalence of *B*. *burgdorferi* in passively collected *I*. *scapularis* was observed in an area where LD is newly emerging [23]. Such findings imply that in areas of long-term hyper-endemicity, annual variation in infection prevalence may not occur and the value of passive surveillance may not be in tracking entomological components of risk as much as gathering data on the when and where of human-tick encounters (i.e., human host focus rather than a tick distribution focus) [12]. For example, our data supports the idea that infected female *I*. *scapularis* are encountered by humans during fall and winter months, underscoring the contribution of this often overlooked stage to human illness [1].

In addition to the utility of passive surveillance for unraveling the human behavioral side of tick-borne disease risk, it can perform a critical service as an early warning system for emerging tick vectors and tick-borne pathogens, as illustrated by several authors [17, 20, 21, 36]. For example, Rand et al. [17] used passive surveillance to demonstrate that *D*. *variabilis* was slowly expanding its range in Maine, while passive surveillance programs in Connecticut, Massachusetts, and Ontario, Canada have identified *A*. *americanum* as an emerging vector in these areas [21, 59, 60]. Additionally, the presence of exotic Asian longhorned ticks (*Haemaphysalis longicornis*) in the United States was only detected after a resident submitted ticks to her local health department [61]. Passive surveillance has the advantage of being less resource intensive than active surveillance, and enables limited funds to be concentrated on direct appraisal of human encounters with ticks.

Relying on public submissions for data collection has limitations. People may not remember or be aware of when the tick was encountered or what they were doing at the time. Submissions depend on public awareness and interest in ticks which can be spatially and temporally variable for reasons that have nothing to do with tick populations, such as media coverage [18, 20, 55]. However, we do not believe that the increasing numbers of submitted *A*. *americanum* (and to a lesser degree *D*. *variabilis*) were a simple reflection of higher public awareness or concern over tick-borne disease risk, otherwise submissions of *I*. *scapularis* would likely have increased as well [17]. They could, however, reflect changing human population density and activities that bring them into contact with *A*. *americanum* and *D*. *variabilis* more frequently than *I*. *scapularis* [21]. The fact that people encounter ticks in an area does not necessarily mean that the area has a high tick density [21] or that those high density areas will remain so in subsequent years [25].

## Conclusions

The results of this study indicate growing importance of vectors other than *I*. *scapularis* in tick-borne disease epidemiology in Monmouth County and New Jersey and, in particular, the greater public health importance of *A*. *americanum* and *D*. *variabilis* in the future [6, 10, 27, 62, 63]. Investigation is required into the role of ticks other than *I*. *scapularis* in the eco-epidemiology of tick-borne disease transmission, focusing on active human disease surveillance; determination of the geographical range of *A*. *americanum*, *D*. *variabilis*, and their associated pathogens; identification of reservoir host(s); determination of transmission efficiency for these etiological agents; and the development of tick management strategies that are effective against both tick species.

Overall, passive surveillance results presented strongly suggest a need to increase public education efforts about the risks for acquiring tick-borne diseases other than LD, including prevention awareness of medically important tick species other than *I*. *scapularis*. Data also demonstrate the need for improved education for particularly vulnerable age groups such as children and the elderly, ensuring prompt tick detection and removal, and preferably, prevention of tick bites altogether. Continued reframing of the conversation about tick exposure risk to include not only risk during outdoor recreational activity but also during more mundane activities like raking leaves or gardening in one’s own backyard, is warranted, as is increased recognition of the role that fall and winter active *I*. *scapularis* females may play in disease transmission.

## Supporting information

S1 Table Ticks acquired in Monmouth County and submitted to Monmouth County Mosquito Control Division’s passive tick surveillance program, 2006–2016. Variables (columns) denoted as follows: IDNO = unique identifier for each tick submission; SPECIES = tick species ID; STAGE = life stage of tick submitted; ENGORG = engorgement level, whether flat (no evidence of blood meal taken) or fed (partially or fully engorged); SUB_COUNTY = submitter’s county of residence; SUB_MUNI = submitter’s municipality of residence; SUB_DATE = date submitted to MCMCD; HOST = host species where tick was found, “NA” designates tick was found unattached; AGE_CLASS = age group of host; SEX = sex of host; ATT_SITE = location on host’s body where tick was attached, “NotAttach” means the tick was not attached; ACQ_LOC = location category where tick was acquired, e.g. home, park, or school; ACQ_MUNI = municipality where tick was acquired; ACQ_CNTY = county where tick was acquired; ACTIVITY = activity the resident was engaged in when tick was acquired; TEST = result of test for *Borrelia burgdorferi*, “NT” = not tested. For all variables “NP” means the resident did not answer this question when filling out the form, and “Unknown” means the resident responded but was unsure of the answer (e.g., if they visited multiple tick-infested locations and so were unable to distinguish where the tick was acquired).
(XLS)
